# CAX‐ing a wide net: Cation/H^+^ transporters in metal remediation and abiotic stress signalling

**DOI:** 10.1111/plb.12460

**Published:** 2016-05-06

**Authors:** J. K. Pittman, K. D. Hirschi

**Affiliations:** ^1^ Faculty of Life SciencesUniversity of ManchesterManchesterUK; ^2^ United States Department of Agriculture/Agricultural Research Service Children's Nutrition Research CenterBaylor College of MedicineHoustonTXUSA

**Keywords:** Abiotic stress, biofortification, Ca^2+^/H^+^ exchanger, CAX structure, phytoremediation, transporter evolution

## Abstract

Cation/proton exchangers (CAXs) are a class of secondary energised ion transporter that are being implicated in an increasing range of cellular and physiological functions. CAXs are primarily Ca^2+^ efflux transporters that mediate the sequestration of Ca^2+^ from the cytosol, usually into the vacuole. Some CAX isoforms have broad substrate specificity, providing the ability to transport trace metal ions such as Mn^2+^ and Cd^2+^, as well as Ca^2+^. In recent years, genomic analyses have begun to uncover the expansion of CAXs within the green lineage and their presence within non‐plant species. Although there appears to be significant conservation in tertiary structure of CAX proteins, there is diversity in function of CAXs between species and individual isoforms. For example, in halophytic plants, CAXs have been recruited to play a role in salt tolerance, while in metal hyperaccumulator plants CAXs are implicated in cadmium transport and tolerance. CAX proteins are involved in various abiotic stress response pathways, in some cases as a modulator of cytosolic Ca^2+^ signalling, but in some situations there is evidence of CAXs acting as a pH regulator. The metal transport and abiotic stress tolerance functions of CAXs make them attractive targets for biotechnology, whether to provide mineral nutrient biofortification or toxic metal bioremediation. The study of non‐plant CAXs may also provide insight into both conserved and novel transport mechanisms and functions.

## Introduction

Ion transporters allow cellular metal homeostasis and drive cytosolic Ca^2+^ signalling, both of which are essential components in the adaptive responses of plants to environmental perturbation. One class of transporter protein that mediates the vectorial transport of both Ca^2+^ and other metal ions is the Cation/H^+^ Exchanger (CAX), a secondary energised transporter that is dependent on a proton (H^+^) gradient across a membrane, and usually localised at an acidic compartment such as the vacuole (Schumaker & Sze 1985; Blumwald & Poole 1986). As H^+^‐coupled antiporters, CAXs are able to mediate the movement of a cation against its concentration gradient and out of the cytosol. Thus it has been proposed that a primary cellular function of the CAX proteins is to restrict cytosolic accumulation of certain free metal ions, such as Mn^2+^ or Cd^2+^ to provide tolerance against metal toxicity, and to maintain a low cytosolic resting concentration of Ca^2+^ to prevent Ca^2+^ toxicity and prime the generation of cytosolic Ca^2+^ signals (Hirschi *et al*. 2000; McAinsh & Pittman 2009). Although CAXs are just one class of ion transporter able to mediate such housekeeping functions, a wealth of evidence now indicates their importance to plants, especially during abiotic stress.

Since the first cloning of plant CAX genes in the mid‐1990s (Hirschi *et al*. 1996), many studies have examined the biochemical, genetic and physiological functions of these transport proteins, principally for isoforms from *Arabidopsis thaliana* and rice (*Oryza sativa*), and often by using the approaches of yeast heterologous expression and T‐DNA insertion mutagenesis (for review see Shigaki & Hirschi 2006; Manohar *et al*. 2011a). Such research began to understand CAXs with regard to their cation transport specificities and kinetics, their importance in mediating metal homeostasis and their potential role in Ca^2+^ signalling. In the last few years, the study of CAX genes has expanded: increasing availability of genome sequences and phylogenetic analyses has identified the wide diversity of CAXs within the green lineage and non‐plant species; high‐resolution protein structural characterisation has begun; and analytical tools such as synchrotron‐based X‐ray fluorescence (SXRF) imaging techniques discern the functions of CAXs in metal homeostasis and cellular partitioning in greater detail. Recent studies showcase the unexpected breadth of physiological and cellular functions that CAXs possess beyond Ca^2+^ homeostasis and new insights are being gleamed from both non‐*Arabidopsis* and non‐plant studies. This review will summarise some of these recent insights, with a focus on the roles of CAXs in abiotic stress responses and potential utilisation for biotechnology.

## Phylogenetic Diversity but Structural Conservation of CAX Proteins

**Figure 1:**
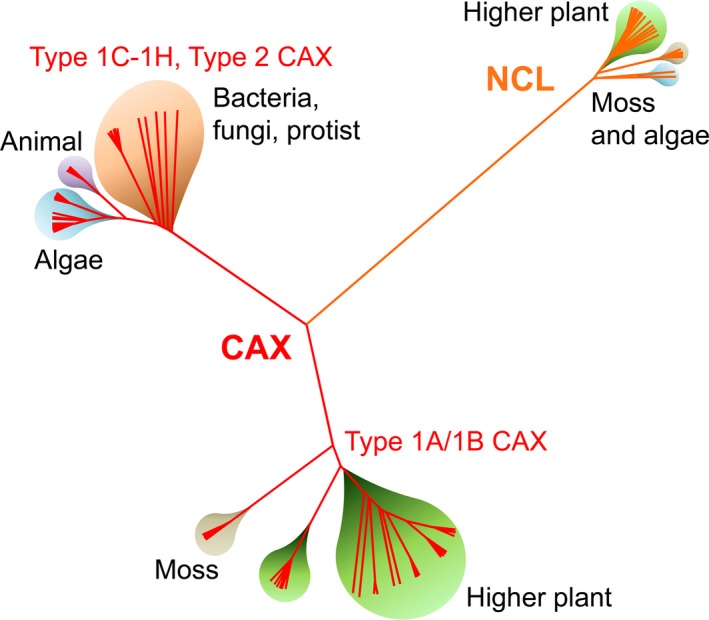
Diversity and expansion of cation/H^+^ exchanger (CAX) and the CAX‐related Na^+^/Ca^2+^ exchanger‐like (NCL) genes in plant and non‐plant organisms. The schematic phylogenetic tree was derived from an analysis of 120 CAX genes and 34 NCL genes from plant, algal, fungal, protist, animal and prokaryotic sequences of Emery *et al*. (2012). The Type 1A‐H and Type 2 CAX sub‐groups are indicated using the definitions as described in Shigaki *et al*. (2006).

Cation/H^+^ Exchangers, along with the functionally equivalent Na^+^/Ca^2+^ exchangers (NCXs) that predominate in animals, are members of the ancient Ca^2+^/Cation Antiporter (CaCA) superfamily of ion‐coupled transporters. The CAXs are a widespread member of this superfamily, present in many bacterial and cyanobacterial species, and in fungi and protists, often as single copy genes (Shigaki *et al*. 2006; Emery *et al*. 2012). CAXs have also been found in some invertebrates and vertebrates, but absent in virtually all mammals. However, it is throughout the green lineage that CAX genes have expanded and diversified (Fig. 1). CAXs are found in many algae, particularly within green algae, and within early land plants such as the lycophyte spikemoss *Selaginella moellendorffii* and the bryophyte moss *Physcomitrella patens* (Emery *et al*. 2012). Two or more CAX genes are seen within some of these photosynthetic organisms, but it is within the higher land plants that CAX genes are present as larger multi‐gene families. On average 5–6 CAX genes per species are found in monocots and dicots, and some species like soybean (*Glycine max*) have 14 CAXs (Emery *et al*. 2012; Pittman & Hirschi 2016). It is still not fully clear whether this expansion in gene family number has led to CAX functional diversity. Ca^2+^ transport function appears to be a conserved characteristic of all CAX genes from all species, where studied, but a broad cation substrate specificity and an ability to transport trace metal ions is a characteristic of some plant CAXs (see below), but generally not for non‐plant CAXs.

The divergence of CAX genes within plants and algae has also led to the presence of CAX‐related genes, which share some phylogenetic and structural characteristics with CAXs but possess one or more EF‐hand‐type Ca^2+^ binding domains (Emery *et al*. 2012; Fig. 1). It has been confirmed that an *Arabidopsis* member of this EF‐hand‐containing group has Ca^2+^ binding activity and intriguingly has vacuolar‐localised Na^+^/Ca^2+^ exchange activity, to provide Na^+^ sequestration in exchange of Ca^2+^ release from the vacuole; hence this novel transporter family has been named Na^+^/Ca^2+^ exchanger‐like (NCL; Wang *et al*. 2012; Li *et al*. 2016). While it is arguable that NCL genes form a distinct CaCA gene family, despite the Na^+^/Ca^2+^ transport characteristics, phylogenetically NCLs group closer to CAX genes than *bona fide* NCX genes (Pittman & Hirschi 2016).

**Figure 2:**
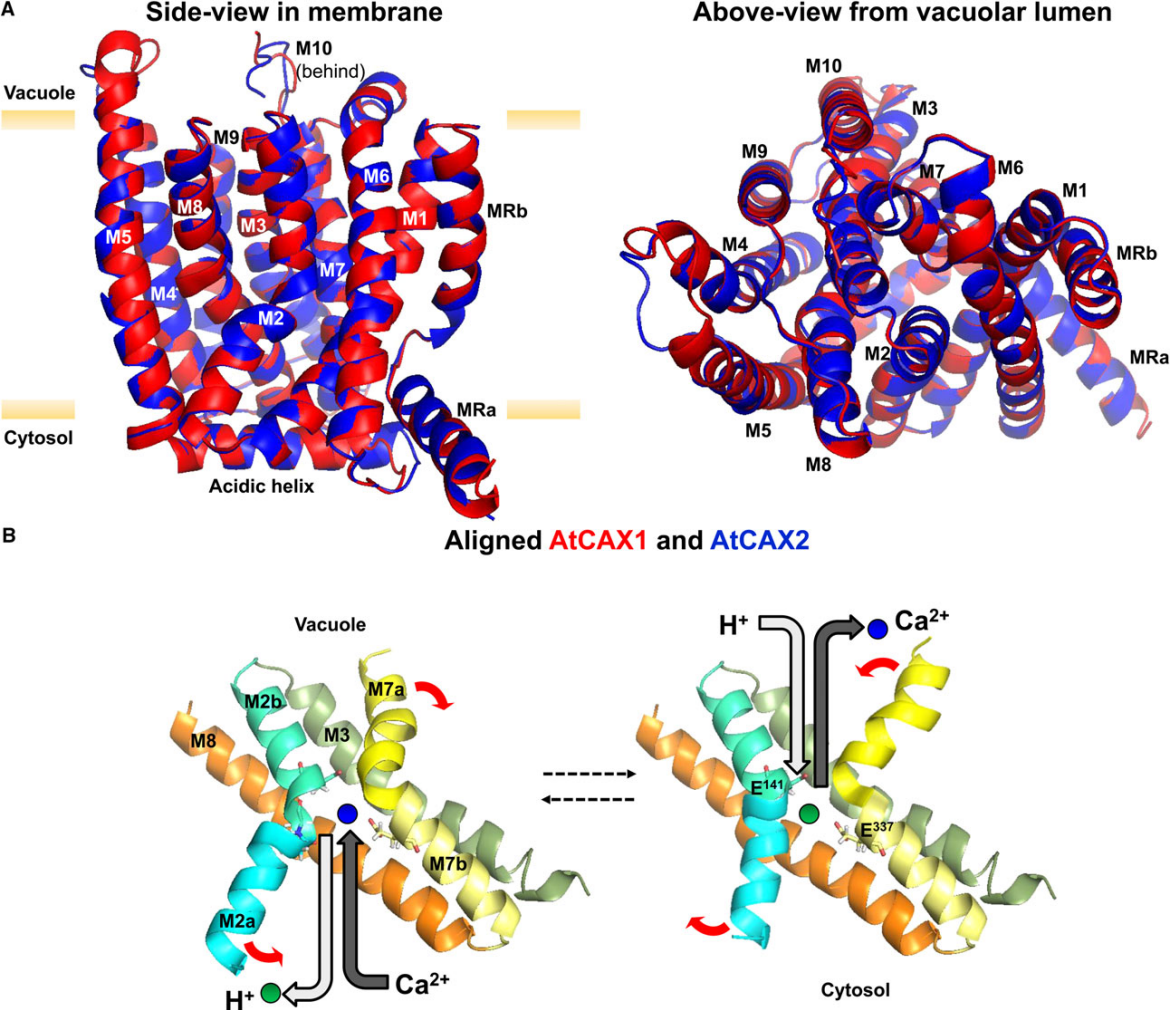
Protein structure models of CAX. A: Structural similarity of *Arabidopsis* CAX1 and CAX2 as determined by homology modelling using the known structure of yeast VCX1 as described in Pittman & Hirschi (2016). CAX proteins have 11 predicted transmembrane (TM) helices (M1–M10) with the first non‐conserved TM labelled as MR (MRa and MRb). B: Model of Ca^2+^ and H^+^ exchange as proposed by Waight *et al*. (2013) and Wu *et al*. (2013). The M2/M3 and M7/M8 helices (from AtCAX1) that derive the conserved α1‐ and α2‐repeat regions, respectively, together make up the cation binding pocket. The M2 and M7 helices are kinked, with each helix half referred to as ‘a’ and ‘b’. Conserved glutamate residues in M2 and M7 that are proposed to bind Ca^2+^ are shown. The proposed movement of cations is indicated with grey arrows. Red arrows indicate protein helix movement that alters conformation of the binding pocket and allows movement of Ca^2+^ across the membrane.

The CAX proteins, along with all CaCA proteins, have a conserved core structure of ten transmembrane (TM) helices, with some of these helices forming highly conserved cation‐binding regions, referred to as α1‐ and α2‐repeat regions within TM helices 2–3 and 7–8, respectively (Cai & Lytton 2004; Fig. 2). A substantial recent advance within CAX research has been the determination of detailed high‐resolution crystal structures of CAX proteins from yeast (*Saccharomyces cerevisiae*; Waight *et al*. 2013), as well as from bacterial CAXs (Nishizawa *et al*. 2013; Wu *et al*. 2013). Such structural information allows a much more detailed insight into the structure–function relationships of CAX proteins and the mechanisms of Ca^2+^/H^+^ exchange. For example, these structures have indicated that the binding of a Ca^2+^ ion and a H^+^ are mutually exclusive within the binding pocket defined by the α‐repeat regions. This suggests a one H^+^ in, one Ca^2+^ out exchange mechanism, with the H^+^ binding, potentially ‘resetting’ the protein structure to allow further Ca^2+^ entry and binding from the other side of the membrane, to continue the cycle (Nishizawa *et al*. 2013; Waight *et al*. 2013; Wu *et al*. 2013; Fig. 2B).

Another insight from these studies is the high structural conservation amongst these CAX proteins from yeast (VCX1), *Bacillus subtilis* (YfkE) and *Archaeoglobus fulgidus* (AfCAX). This structural conservation provides the expectation that plant CAX protein structure is also highly conserved and allows use of these non‐plant structures to gain insight into plant CAX structure–function. For example, the VCX1 structure can be used as a template for protein homology modelling to predict plant CAX structure, as recently performed for rice OsCAX1a (Pittman & Hirschi 2016), and shown here for *Arabidopsis* CAX1 and CAX2 (Fig. 2A). All of these structures show high similarity, and include 10 conserved TM helices, plus a further ‘redundant’ N‐terminal transmembrane helix and a cytosolic acidic helix. The α‐repeat structures are also highly conserved. A further observation from the CAX structural studies was that the crystals either contained homomeric CAX dimers (for VCX1 and AfCAX) or trimers (for YfkE) (Nishizawa *et al*. 2013; Waight *et al*. 2013; Wu *et al*. 2013). CAXs from plants have previously been reported to form homomeric or indeed heteromeric oligomers, with potential implications for functional activity (Zhao *et al*. 2009a,b). Thus structural information may be able to provide further insight into the relevance of CAX complexes in the future.

## The CAX Transporters in Abiotic Stress Signalling

Cytosolic Ca^2+^ and metal homeostasis are important components in adapted biological responses. A component of this regulation is the plant sequestration of ions into the vacuoles (Pittman 2011; Gao *et al*. 2015). CAXs appear to be a fulcrum for modulating ion homeostasis, pH and redox levels within plant cells (Conn *et al*. 2011; Cho *et al*. 2012).

Yeast is a viable model for the study of CAX function under various stresses. Fungi and plants have similar ion transport systems at their vacuolar membranes (Yesilirmak & Sayers 2009). Furthermore, fungi often possess just one or two CAX genes, affording ease of genetic analysis. Thus studies of yeast VCX1 function in response to abiotic stress responses may provide useful insight. For example, hypertonic shock, such as high NaCl, KCl or sorbitol addition, induces a cytosolic Ca^2+^ burst, and VCX1 is required to rapidly remove this Ca^2+^ (Denis & Cyert 2002). Likewise, yeast studies clearly demonstrate that VCX1 is able to rapidly sequester Ca^2+^ into the vacuole during a calcium (Ca) stress episode (Miseta *et al*. 1999). Furthermore, the transporter appears to impact pH homeostasis both in the vacuolar lumen and cytosol (Papouskova *et al*. 2015). Impairing VCX1 function, but not the vacuolar Ca^2+^‐ATPase PMC1, makes yeast cells tolerant to cadmium (Cd) stress conditions (Ruta *et al*. 2014). This implies that VCX1 may participate in metal tolerance *via* modulation of the cytosolic Ca^2+^ response rather than through direct Cd^2+^ transport.

**Figure 3:**
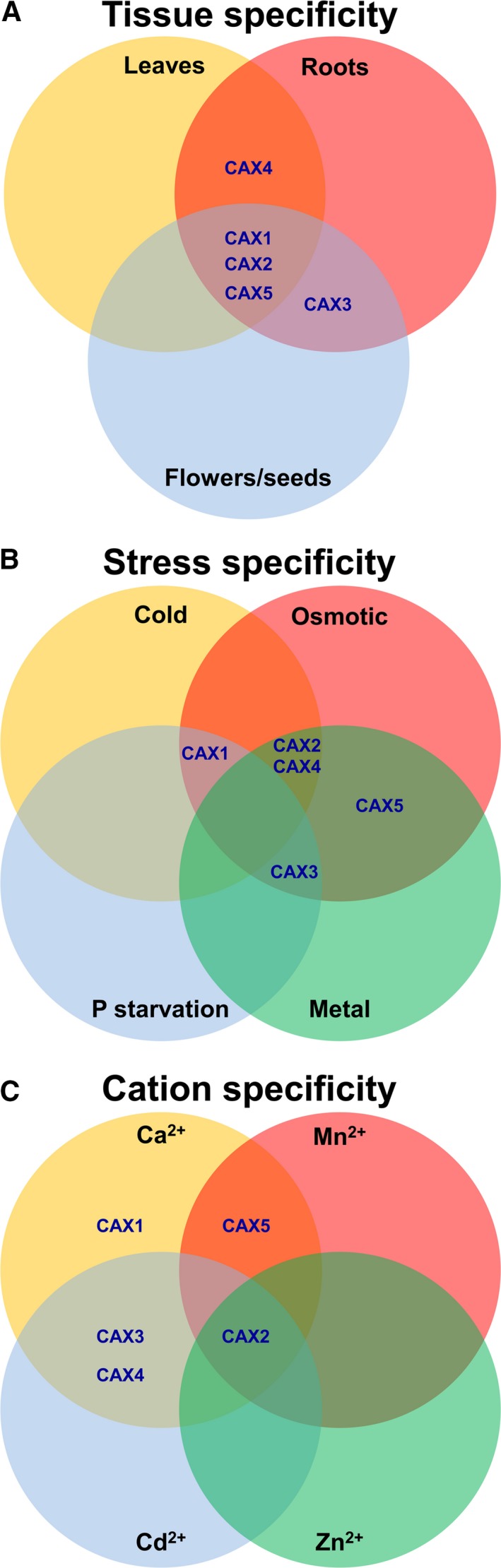
Diversity and overlap of tissue specificity (A), stress response pathway specificity (B) and cation substrate specificity (C) for five *Arabidopsis* CAXs.

Cross‐talk among members of the CAX gene family in plants along with compensatory mechanisms account for subtleties in phenotypes associated with loss of function of single family members (Cheng *et al*. 2005; Connorton *et al*. 2012; Fig. 3). Although progress using yeast heterologous expression has delineated CAX transport properties, this approach has limitations in terms of discriminating CAX functions (Shigaki & Hirschi 2000; Manohar *et al*. 2011b). This has often led to ascribing *in planta* function to CAXs based on ectopic expression and knockout mutant analysis. These types of studies have shown the importance of CAX in maintaining Ca^2+^ homeostasis in plants. In tobacco (*Nicotiana tabacum*) plants over‐expressing a deregulated (N‐terminal truncated) *Arabidopsis* CAX, Ca^2+^ accumulation is 30% higher in leaves compared with a vector control, and double in the roots (Hirschi 1999; Mei *et al*. 2007). These lines show a number of symptoms similar to Ca^2+^ deficiencies, and tomato lines expressing the same deregulated CAX suffer from blossom end rot (Park *et al*. 2005; De Freitas *et al*. 2014). This relatively common garden problem is a physiological disorder caused by a Ca^2+^ imbalance within the plant (De Freitas *et al*. 2014). In mesophyll cells, CAXs appear to be especially important in maintaining Ca^2+^ homeostasis as well as stomatal conductance (Conn *et al*. 2011). These phenotypes suggest that altered CAX expression misappropriates Ca^2+^ within plant cells. Furthermore, when CAX function is diminished in *Arabidopsis cax1* lines, the mutants are sensitive to high levels of magnesium (Mg^2+^) and grow better in serpentine condition with a low Ca^2+^/Mg^2+^ ratio (Bradshaw 2005).

When two highly expressed CAX transporters are mutated in *Arabidopsis* the phenotype is dramatic (Cheng *et al*. 2005). The Ca^2+^ content is reduced but so is Mg^2+^, while phosphorus (P), Mg^2+^ and zinc (Zn) levels are increased. This phenotype epitomises the challenges of working with ‘promiscuous’ transporters that broadly alter the ionome. Are the stunted growth, leaf tip necrosis and small siliques of the *cax1cax3* double mutant a function of impaired Ca^2+^ homeostasis or alterations in the concentration of other metals? The P phenotype of this double mutant suggest that CAX transporters alter root–shoot signalling during phosphate starvation (Liu *et al*. 2011). Furthermore, transcriptomic microarray data of the double mutant further document what one would expect, a multitude of changes in gene expression (Conn *et al*. 2011), thus further challenging the mechanistic interpretation of these phenotypes.

In addition to yeast heterologous expression and genetic mutation of CAX genes, genome‐scale transcriptomic analysis can provide information about the relative expression changes of CAX transcripts in response to abiotic stresses; for example, many *Arabidopsis* and rice CAX transcripts are increased or decreased in abundance in response to water deficit stresses, including drought, heat, cold or salinity (summarised in Bickerton & Pittman (2015)). Inducing enhanced CAX expression in plants also suggests that these transporters have a role in salt stress response (Shigaki & Hirschi 2006; Manohar *et al*. 2011a). Studies in rice, tobacco, soybean and the halophyte *Suaeda salsa* support this observation. For example, expression of rice CAX genes including *OsCAX4* is high in response to prolonged salt stress (Yamada *et al*. 2014). Ectopic expression of *SsCAX1* in *Arabidopsis* and *AtCAX1* in tobacco increases salt sensitivity (Mei *et al*. 2007; Han *et al*. 2012). This suggests that CAX activity must be carefully modulated during a salt stress episode. Expression of CAX transcripts in *Arabidopsis* plants subjected to salt stress suggests a complex network of changes among the transporters (http://bar.utoronto.ca/efp/cgi-bin/efpWeb.cgi). For example, *AtCAX1* expression decreases in aerial tissues while *AtCAX3* expression is enhanced in the roots. Meanwhile, increased expression of a soybean CAX (*GmCAX1*) increased salt tolerance of the plant. Interestingly, *GmCAX1* is induced by various stress treatments, and the protein is proposed to localise to the plasma membrane (Luo *et al*. 2005). However, caution must be used in basing functional analysis of transporters on their mRNA abundance (Vogel & Marcotte 2012) and ectopic expression phenotypes (Diener & Hirschi 2000). The first direct evidence of Na^+^ transport mediated by a CAX from a photosynthetic organism came from work in the green alga *Chlamydomonas reinhardtii* (Pittman *et al*. 2009), yet Na^+^ transport activity for a higher plant CAX has not been seen, apart from vacuolar Na^+^ sequestration *via* Na^+^/Ca^2+^ exchange by AtNCL (Li *et al*. 2016).

During a salt stress episode, CAXs may function in regulating pH levels. Generation of changes in cellular pH with specific amplitude and duration around the vacuole may play a vital role for activating the defence response mechanisms for salinity tolerance (Kader & Lindberg 2010). CAXs can be regulated by cytosolic pH (Pittman *et al*. 2005), and evidence suggests altered regulation of proton pumps when CAX expression is deregulated (Zhao *et al*. 2008; Conn *et al*. 2011). Evidence has also linked CAX activity with apoplastic pH homeostasis in guard cells (Cho *et al*. 2012). This close relationship observed between CAX and pH may signify a role in modulating endomembrane pH levels similar to the pH regulation by NHX‐type antiporters required for protein trafficking (Pittman 2012; Reguera *et al*. 2015).

In *Arabidopsis* and cotton (*Gossypium hirsutum*), studies suggest that CAXs play a role in cold tolerance (Catalá *et al*. 2003; Xu *et al*. 2013). It is noteworthy that in *Arabidopsis* a single CAX transporter (AtCAX1) appears to be required for freezing tolerance after cold acclimation, while the studies in cotton highlight the importance of a different CAX transporter, in this case GhCAX3, in the cold acclimation response. This suggests that species may utilise different CAXs and signalling pathways to transduce the cold signal. Different CAX isoforms have also been implicated in the response of roots to hypoxia, often caused by waterlogging (Wang *et al*. 2016). An increase in Ca^2+^ accumulation is observed in response to hypoxic treatment, and this Ca^2+^ stress is proposed to be mitigated by specific CAX isoform function in *Arabidopsis*. However, in this response pathway, CAX proteins appear to function in tandem with ACA‐type Ca^2+^‐ATPases (Wang *et al*. 2016).

In addition to a role of CAXs in response to abiotic stresses such as salinity and cold that are clearly regulated *via* cytosolic Ca^2+^ signal modulation, CAXs are also implicated in toxic metal stress adaptation. While some CAXs can mediate trace metal ion transport, and therefore potentially provide tolerance to metal stress via direct metal sequestration (Hirschi *et al*. 2000; Korenkov *et al*. 2007; Edmond *et al*. 2009; Migocka *et al*. 2011), it is unclear whether all examples of CAX involvement are due to direct metal transport rather than a Ca^2+^ signalling role. For example, expression data indicate the involvement of CAXs in boron (B) deficiency responses (Quiles‐Pando *et al*. 2013; Gonzalez‐Fontes *et al*. 2014), but are the CAXs involved in conveying the B deficiency or the associated Ca^2+^ signal? Cautious interpretation must be employed as a wide range of abiotic stresses appear to reduce or enhance CAX expression (Bickerton & Pittman 2015).

Another example of this is with regard to metal‐tolerant plants that hyperaccumlate Cd^2+^ and display altered CAX expression (Baliardini *et al*. 2015; Zhang *et al*. 2016). A CAX2‐like transporter isolated from *Sedum alfreddi*, a Zn/Cd hyperaccumulating plant, when expressed in tobacco causes enhanced Cd^2+^ accumulation (Zhang *et al*. 2016). In a metal hyperaccumlating *Arabidopsis halleri* plant grown under low Ca^2+^ conditions, a specific CAX gene (*AhCAX1*) appears to attenuate the metal stress (Baliardini *et al*. 2015). Furthermore, the predominately root‐specific *AtCAX4* of *Arabidopsis* is expressed at high levels during a Cd^2+^ stress (Mei *et al*. 2009). Accumulation of Cd^2+^, like many abiotic stresses, produces reactive oxygen species (ROS) that then generate alterations in cytosolic Ca^2+^ levels (Garnier *et al*. 2006; Ismail *et al*. 2014). Particular CAX transporters appear to attenuate ROS. In barley (*Hordeum vulgare*) lines exposed to Cd^2+^, changes in CAX expression have been confirmed by analysis of the tonoplast proteome (Schneider *et al*. 2009). These studies suggest direct CAX‐mediated Cd^2+^ transport of the metal ion into the plant vacuole; however, direct transport experiments are needed to substantiate these claims.

## The CAX Transporters in Biotechnology

The nutrients in the human diet ultimately come from plants. Manipulating plant transporters may be a means to mine specific nutrients out of the soil into the edible portion of foods (Conn *et al*. 2012). The overexpression of CAXs is a proven strategy for altering Ca^2+^ and trace mineral content in numerous crops (Manohar *et al*. 2011a; Robertson 2013). Potatoes, carrots, lettuce and tomatoes have increased levels of Ca^2+^ content in the edible portions of the food through enhanced CAX expression (Hirschi 2008). Using stable isotope labelling methods and human subjects the CAX‐enhanced carrots appear to contain more bioavailable Ca^2+^ (Morris *et al*. 2008). Furthermore, using a panel of taste testers, CAX expressing lettuce varieties appear to have retained their taste and texture qualities (Park *et al*. 2009). Recent genetic and comparative genomic analysis has also confirmed the utility of altering CAX expression in *Brassica rapa* for altered Ca^2+^ composition and concentration (Graham *et al*. 2014).

The benefits of CAX expression in transgenic plants may not be limited to the edible portions of foods or merely for nutritional improvement. Enhanced expression of CAX in gourd rootstocks grafted to watermelon can also improve fruit quality (Han *et al*. 2009). Likewise, *in silico* expression analyses has revealed that CAX genes are highly expressed in the initial stages of seed development in rice (Goel *et al*. 2012). This suggests that enhanced expression of CAX genes may improve rice varieties (Kim *et al*. 2005).

Enhanced and manipulated CAX expression may provide tolerance to metal stress in plants through the vacuolar sequestration of Cd^2+^, Mn^2+^ and Zn^2+^ (Manohar *et al*. 2011a; Socha & Guerinot 2014). Specific CAX1 mutant variants increase metal transport in yeast expression assays while dramatically decreasing Ca^2+^ transport (Shigaki *et al*. 2005). These mutants provide tools for redistributed metals across plant membranes. The utility of CAX engineering is demonstrated when a CAX with higher Cd^2+^ transport rates was over‐expressed in petunia (*Petunia* *×* *hybrida*) plants to confer increased tolerance to the metal (Wu *et al*. 2011). Improved sequestration of metals such as Cd^2+^ in the root tissues of plants can reduce Cd^2+^ levels in edible tissues, as Cd^2+^ exposure is a major hazard to human health (Nawrot *et al*. 2010). Root‐specific expression of CAX transporters mitigates Cd^2+^ content in tobacco leaves (Korenkov *et al*. 2007, 2009). Future work may also utilise CAX from microalgae such as *Chlamydomonas* to remediate Cd^2+^‐polluted freshwater sites (Pittman *et al*. 2009).

**Figure 4:**
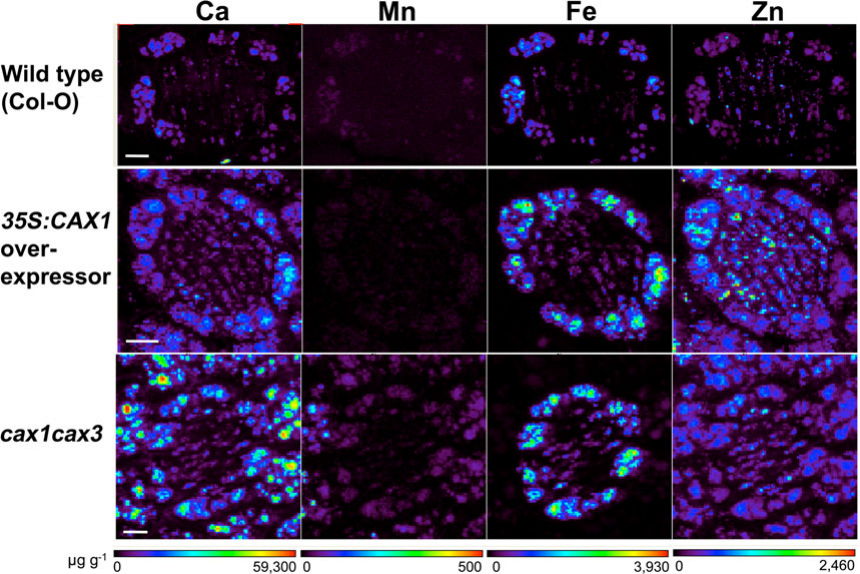
Changes in nutrient partitioning in *Arabidopsis* embryos in response to CAX manipulation. High resolution mapping of Ca, Mn, Fe and Zn in wild type, 35S:sCAX1 overexpression and *cax1cax3* knockout mutant embryo sections. Derived from data described in Punshon *et al*. (2012).

To date, understanding of CAX genetic manipulation for either biofortification or bioremediation has lacked a clear understanding of the changes in metal partitioning that occur at the cellular level. The use of preparation‐free elemental imaging technologies such as SXRF microscopy is beginning to allow researchers to understand the relationship between CAX expression and nutrient bioavailability or metal partitioning (Punshon *et al*. 2009; Yang *et al*. 2012). For example, SXRF has already been used to investigate changes in distribution and abundance of Ca^2+^ and other trace metals attributable to the vacuolar membrane antiporters in *Arabidopsis*. Images show that perturbed CAX activity alters the Ca^2+^ partitioning within cells of the developing embryo (Punshon *et al*. 2012; Fig. 4).

Modulating CAXs could also be an important pharmaceutical target to limit human or animal parasite transmission (Kmetzsch *et al*. 2011). For example, apicomplexan parasites that cause malaria or toxoplasmosis (*e.g*. *Plasmodium* sp. and *Toxoplasma gondii*) utilise CAXs to help development within the mosquito midgut (Guttery *et al*. 2013). The transporter is essential to protect these parasites against extracellular Ca^2+^ levels in the gut, and thus inhibiting this transporter could provide a target for disease eradication.

## Non‐Plant CAXs – Lessons to be learned?

Apart from yeast VCX1, the vast majority of extensively characterised CAXs have been from higher plants, and mostly from *Arabidopsis* and rice. Genomics and phylogenetic analyses are increasingly demonstrating that CAX genes are found throughout life (Fig. 1), as described above, yet most plant and non‐plant CAX genes remain uncharacterised. Recent studies are beginning to investigate the functions of CAX genes from various non‐plant organisms, including animals. Parasite CAXs, such as from the malarial parasites *Plasmodium*, share phylogenetic and functional similarities with higher plant and algal CAXs (Pittman *et al*. 2009; Guttery *et al*. 2013), yet the subcellular CAX localisation appears to differ, despite apicomplexan parasites possessing plant‐like vacuoles. CAX from *Plasmodium falciparum* was suggested to be mitochondrial (Rotmann *et al*. 2010), while CAX from the related *Plasmodium berghei* was observed at an unknown endomembrane, possibly a secretory pathway location (Guttery *et al*. 2013), indicating that CAXs with very different subcellular locations in some species may exhibit very different physiological functions. One possible function of these CAXs is for Ca^2+^ sequestration into an intracellular compartment to provide cellular Ca^2+^ tolerance, equivalent to the role of CAX in yeast and plants. In contrast, it was suggested that PfCAX functions in Ca^2+^ efflux from the inner mitochondrial matrix (Rotmann *et al*. 2010), a function performed by a CCX‐type Na^+^/Ca^2+^ exchanger in animals (Palty *et al*. 2010), but by a less clear mechanism in plants (Wagner *et al*. 2016). Whether apicomplexan CAXs are important for modulating Ca^2+^ signals is unknown. The phenotype of *pbcax* mutants suggests a predominant role in providing Ca^2+^ tolerance (Guttery *et al*. 2013), but it is possible that the developmental defect of *pbcax* parasites may be partly due to impaired Ca^2+^ signalling.

The CAX genes have been identified in various animals, including fish, amphibians, reptiles, birds and some mammals, but not placental mammals (Melchionda *et al*. 2016). The first animal CAX to be functionally examined was a zebrafish (*Danio rerio*) CAX, shown to be required for neural crest development, potentially through modulation of pH and Ca^2+^ homeostasis (Manohar *et al*. 2010). Subsequently, a frog (*Xenopus laevis*) CAX has also been found expressed in neural crest cells and required for cell migration, by controlling the formation of stable focal adhesions during cell spreading (Melchionda *et al*. 2016). Moreover, this study demonstrated that the animal CAX, localised at acidic lysosomes, regulates cytosolic Ca^2+^ signals. This work clearly demonstrates that animal CAX proteins are instrumental for Ca^2+^ loading into lysosomes, which are being recognised as an important intracellular Ca^2+^ signalling store in mammalian cells (Lloyd‐Evans 2016). It is unclear whether CAXs are essential for the function and Ca^2+^ loading of acidic organelles in other species but they are not involved in this process in higher mammals, including humans, as CAX genes were apparently lost in the higher mammalian lineage (Emery *et al*. 2012; Melchionda *et al*. 2016). For plant CAXs, direct observation and confirmation of CAX‐dependent Ca^2+^ signal modulation has so far been challenging. Therefore the frog CAX study provides compelling evidence that CAX proteins can be considered as important components of Ca^2+^ signal generation, rather than just cellular ‘housekeepers’ to maintain Ca^2+^ homeostasis.

## Conclusions

While CAX proteins in plants have long been recognised as important ‘housekeeping’ components in cellular Ca^2+^ and trace metal homeostasis (Hirschi *et al*. 1996, 2000; Ueoka‐Nakanishi *et al*. 1999), recent studies are beginning to implicate CAXs in a range of cellular and physiological processes, including stomatal function *via* cellular pH regulation, phosphate starvation signalling and hypoxia. One of the challenges ahead is to discern the exact mechanism of CAX function in each of these pathways, whether by direct modulation of a cytosolic Ca^2+^ ‘signature’ (McAinsh & Pittman 2009), *via* modulation of a pH signal (Pittman 2012) or in some cases *via* the direct transport of an alternative substrate, such as Na^+^ (Luo *et al*. 2005). Furthermore, many apparent functions of CAX isoforms, as indicated by reverse genetics approaches, may be an indirect consequence of altered metal homeostasis. Because even quite subtle changes in cytosolic Ca^2+^ dynamics can yield substantial changes in downstream gene expression (Whalley & Knight 2013; Liu *et al*. 2015), gross modifications to cellular Ca^2+^ such as might be caused by knockout or overexpression of a Ca^2+^ efflux transporter like a CAX, may induce gene expression changes and result in a plant phenotype that are not necessarily due to specific CAX function. However, the observations that different CAX isoforms appear to be involved in distinct and specific plant processes does provide indication of direct CAX involvement in some abiotic stress pathways.

Finally, evidence from non‐plant studies are beginning to provide confirmation that CAX proteins are indeed able to modulate Ca^2+^ signals in order to mediate a specific signalling outcome. The advantage of many of these non‐plant CAX models, such as *Xenopus* CAX (Melchionda *et al*. 2016) or *Plasmodium* CAX (Guttery *et al*. 2013), is that the CAXs are encoded by single genes in these species and therefore genetic dissection of CAX Ca^2+^ signalling is not hampered by genetic compensation and redundancy challenges. Furthermore, the study of plant CAXs may be further challenged by the potential of homomeric and heteromeric CAX complexes exhibiting different functional properties (Zhao *et al*. 2009a,b). Therefore, the study of plant CAXs may require more imaginative approaches going forward.
